# Spontaneous massive fetomaternal hemorrhage: two case reports and a literature review of placental pathology

**DOI:** 10.1186/s12884-023-05826-9

**Published:** 2023-07-21

**Authors:** Yushuang Zheng, Donglu Li, Xinran Li, Aman Zheng, Fan Wang

**Affiliations:** 1 Departments of Obstetrics and Gynecology, The Second Affiliated Hospital and Yuying Children’s Hospital of Wenzhou Medical University, 109 West Xueyuan Road, Lucheng District, Wenzhou, 325000 China; 2 The Second Clinical Medical College, Wenzhou Medical University, Wenzhou, Zhejiang China

**Keywords:** Anemia neonatorum, Histopathology, Maternal-fetal exchange, Pathology, Placenta

## Abstract

**Background:**

Massive fetomaternal hemorrhage (FMH) is a rare event during pregnancy that may cause severe fetal anemia or death.

**Case presentation:**

This paper reports two cases of fetomaternal hemorrhage with unexplained reasons. Both cases required emergency caesarean sections for non-reassuring fetal status and were treated with neonatal blood transfusion. Fetomaternal hemorrhage was confirmed via maternal Kleihauer-Betke test.

**Conclusion:**

We found parenchymal pallor, increased nucleated red blood cells (nRBCs), and syncytial knots (SKs) in the placentas, which are compatible with fetal anemia. Immunohistochemical staining indicated VEGF, CD34, and CD31 expression in the endothelial cells of the capillaries, characteristic of massive FMH placenta. This article also reviews the particular histopathological changes in FHM placenta according to the placental lesion classification system.

## Background

Fetomaternal hemorrhage (FMH) is fetal blood leak through the damaged placental barrier into the maternal blood circulation that can result in fetal blood loss and maternal hemolytic reactions. Nearly all pregnancies include small volumes of fetal red cells crossing into the maternal circulation without evident clinical effects because of the bidirectional passage of nucleated cells and red blood corpuscles through the placenta [1]. Only when blood loss reaches a significant volume, such as 20% of the fetoplacental blood volume, or a rapid speed can it become symptomatic [2]. Moderate to severe FMH is a less common event, occurring 1–3 times per 1,000 live births [3]. Spontaneous massive FMH is characterized by the transfer of fetoplacental blood volume greater than 20% of the fetal blood volume [4], without any notable antecedent history of trauma, and absence of clinical or histopathological signs of abruption [5]. Most cases of massive FMH are reported to occur during the second or third trimesters [3]. The symptoms and signs of spontaneous FMH at presentation are subtle and prenatal diagnosis is difficult. FHM causes more severe subsequent adverse outcomes, including fetal anemia, neurologic injury, and demise or neonatal death, some of which may affect long-term outcomes [4]. Fetomaternal hemorrhages account for 4.1% of antepartum stillbirths, and a higher proportion of these stillbirths occur at term gestation compared to other causes [6]. Furthermore, the etiology and mechanisms of spontaneous FMH have not been completely illustrated.

As FMH occurs transplacentally, histopathological examination of the placenta may provide useful information for elucidating the pathogenesis of FMH in cases without an obvious cause. Until recently, relatively few studies have evaluated placental pathology in pregnancies complicated by massive FMH. Placental hemorrhage is considered the most common pathological change of FMH. The intervillous thrombi, increased nucleated red blood cells, syncytial knots, parenchymal pallor and placental villi edema are also associated with FMH. Herein we describe two cases of spontaneous massive fetomaternal hemorrhage. Hematoxylin and eosin (H&E) slides were reviewed to identify relevant lesions, and paraffin blocks were stained with endothelial markers (VEGF, CD34, and CD31) with emphasis on angiogenesis. We describe characteristic gross and microscopic findings of these full-term placentas and review the literature to further study the particular placental pathologic and functional changes among pregnancies affected by FMH. Both patients provided written informed consent for publication of this case series and any accompanying images.

## Case presentation

### Clinical finding

#### Case 1

A 31-year-old woman, gravida 2, para 1, presented to the labor and delivery unit complaining of decreased fetal movement for 1 week at 37 + 5 weeks’ gestation. No trauma had occurred. She had a history of one previous normal vaginal delivery and an uncomplicated antenatal course during her current pregnancy. An ultrasonographic investigation showed a singleton fetus with growth measurements corresponding to 37 weeks and no fetal abnormalities or polyhydramnios. The placenta was normal in appearance and location. A cardiotocogram on admission demonstrated an apparent loss of baseline variability. The patient underwent an emergency cesarean section because of non-reassuring fetal status.

At delivery, the male neonate weighed 3,020 g with Apgar scores of 6 at 1 min and 7 at 5 min. His heart rate was 132 beats/min, respiratory rate was 56/min, and blood pressure was 56/32 mmHg. He was extremely pale and his activity was reduced, although he was not hydropic. He needed respiratory support via an oxygen mask. Anemia was revealed by a blood test (red blood cell count, 0.79 × 10^12^/mL; hemoglobin concentration, 2.6 g/dL; and hematocrit, 9%). Liver function tests were as follows: total bilirubin, 9.2 umol/L; aspartate aminotransferase, 43 U/L; alanine aminotransferase, 6 U/L; glucose, 5.1 mmol/L; and C-reactive protein, 8 mg/dL. Initial arterial blood gas revealed metabolic acidosis, with pH, 7.19; pCO_2_, 36 mm Hg; and BE, -13.5. The blood type of both the mother and infant was O rhesus positive. Screening tests for congenital infection were all negative as was maternal parvovirus B19 IgM. The percentage of maternal hemoglobin F (HbF) was very high (4.8%; normal range is < 2.0%), and the maternal serum alpha-fetoprotein level was extremely high (7247 ng/mL; normal range at term pregnancy is < 400 ng/mL). Kleihauer-Betke (KB) testing of the maternal blood sample was positive. Therefore, fetomaternal transfusion was diagnosed based on the aforementioned findings. On postpartum day 21, the percentage of the mother’s serum HbF was 0%.

The newborn was subsequently treated with blood transfusions for a total dose of 155 mL type O packed red cells, 50 ml plasma, and 3 g albumin. He soon responded well. He was discharged following a 7-day hospitalization with a normal follow-up examination.

#### Case 2

A 30-year-old woman in her first pregnancy was transferred from her community hospital at 36 weeks’ gestational age because of suspected fetal growth restriction. She had an uncomplicated antenatal course during pregnancy. An ultrasonographic investigation showed a singleton fetus with mild fetal growth restriction (estimated fetal weight near the tenth percentile, normal amniotic fluid volume, and normal Doppler findings) and no fetal abnormalities. The placenta was normal in appearance and location. The patient reported normal fetal movement. After maternal nutritional supplementation and oxygen therapy for 1 week, she had an emergency cesarean section because an cardiotocogram demonstrated recurrent variable decelerations with minimal variability, indicating a non-reassuring fetal status.

A male infant was born weighing 2,380 g with Apgar scores of 9 at 1 min and 9 at 5 min. The infant’s vital signs on admission were as follows: heart rate, 138 beats/min; respiratory rate, 45/min; blood pressure, 53/31 mmHg; and temperature, 36℃. The neonate was pale without hydrops fetalis. The admission blood test was as follows: red blood cell count, 1.44 × 10^12^/mL; hemoglobin concentration, 5.2 g/dL; and hematocrit, 18%. The liver function tests were as follows: total bilirubin, 6.7 umol/L; aspartate aminotransferase, 59 U/L; alanine aminotransferase, 12 U/L; glucose, 4.5 mmol/L; and C-reactive protein, less than 8 mg/dL. The first arterial blood gas analysis at 15 min was normal. The infant’s blood type was B rhesus positive, whereas that of his mother was A rhesus positive and the Coombs test was negative. Screening tests for congenital infection were all negative as was maternal parvovirus B19 IgM. Fetal anemia secondary to fetomaternal transfusion was confirmed by a maternal KB test.

The infant responded well to intravascular fluid therapy followed by a total dose of 70 mL type B packed red cells and 2.3 g albumin. A neurological examination and intracranial ultrasound were normal, and the infant was discharged in good condition after 4 days.

### Pathological findings

**Fig. 1:**
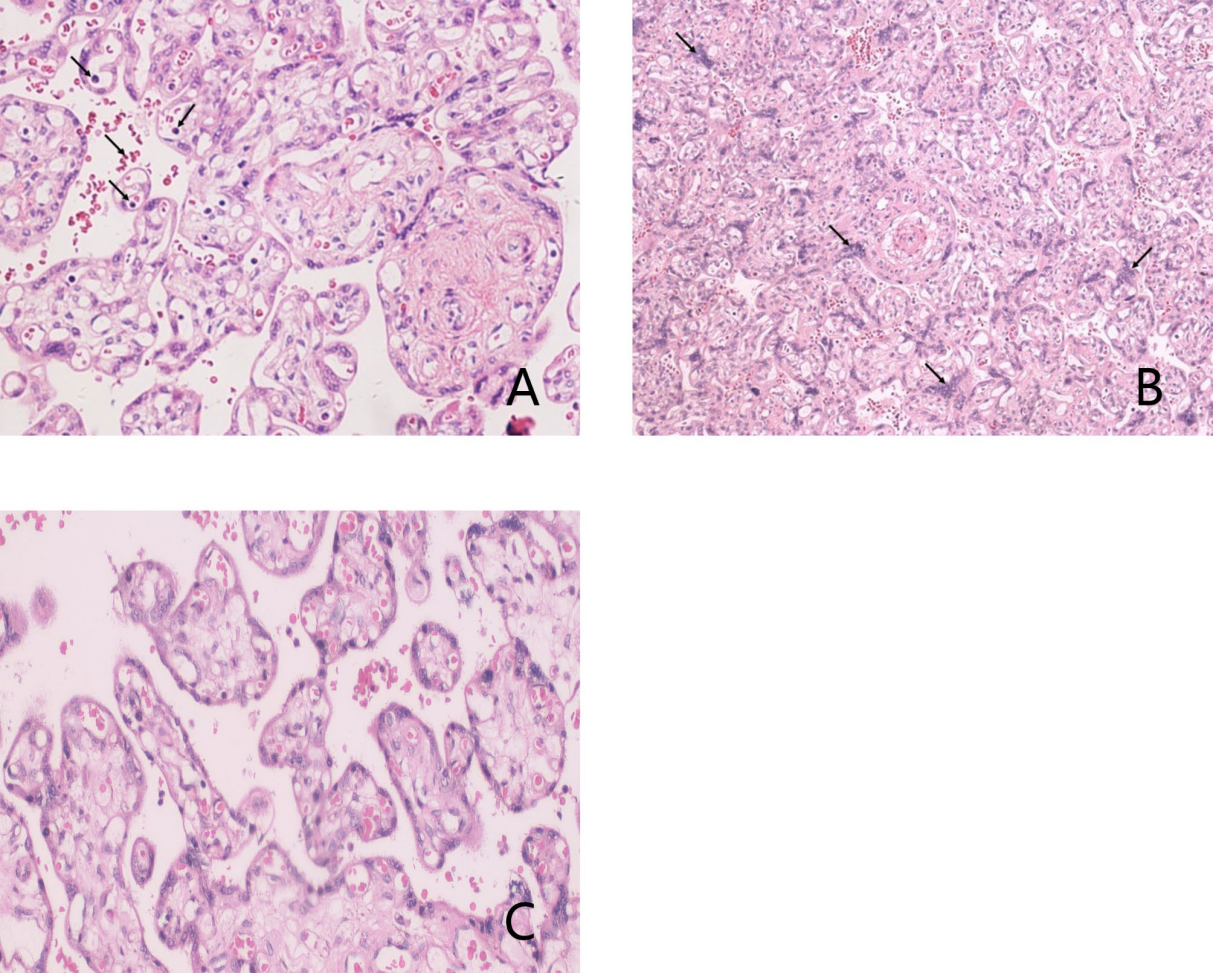
Representative microscopic features of placenta in our case. H&E-stained sections of placentas with mild increased nucleated red blood cells (arrows) within the vessels and intervillous space (**A**). SK (arrows) density was increased in FMH placentas (**B**) as compared to healthy placenta (**C**).(200X original magnification)

The placentas were examined to clarify the mechanisms of fetomaternal transfusion. The two cases’ placentas both had appropriate weights for the gestational age and the fetal/placental weight ratios were normal, but the infants appeared pale. Microscopically, the vessels in the stem villi were dilated, and most of the lumens of these vessels were empty. There was stromal fibrosis and reduced capillaries in the secondary villi. Fibrosis and distention of the capillaries was seen in parts of the terminal villi. The red blood cells were decreased in the lumens. The scattered nucleated red blood cells (nRBCs) were mildly increased within the vessels and placental intervillous space, consistent with hemorrhage originating from fetal circulation (Fig. 1a) [7]. There was increased syncytial knot (SK) density indicating > 1 in 3 to 5 terminal villi and > 10 nuclei per knot in the placenta (Fig. 1b-c). There was also increased perivillous fibrin deposition in some of the remaining intervillous spaces and calcification in the small areas of the parenchyma. Localized hemorrhage was found within the mucous tissue. The blood vessels in the umbilical cord were normally developed.

**Fig. 2:**
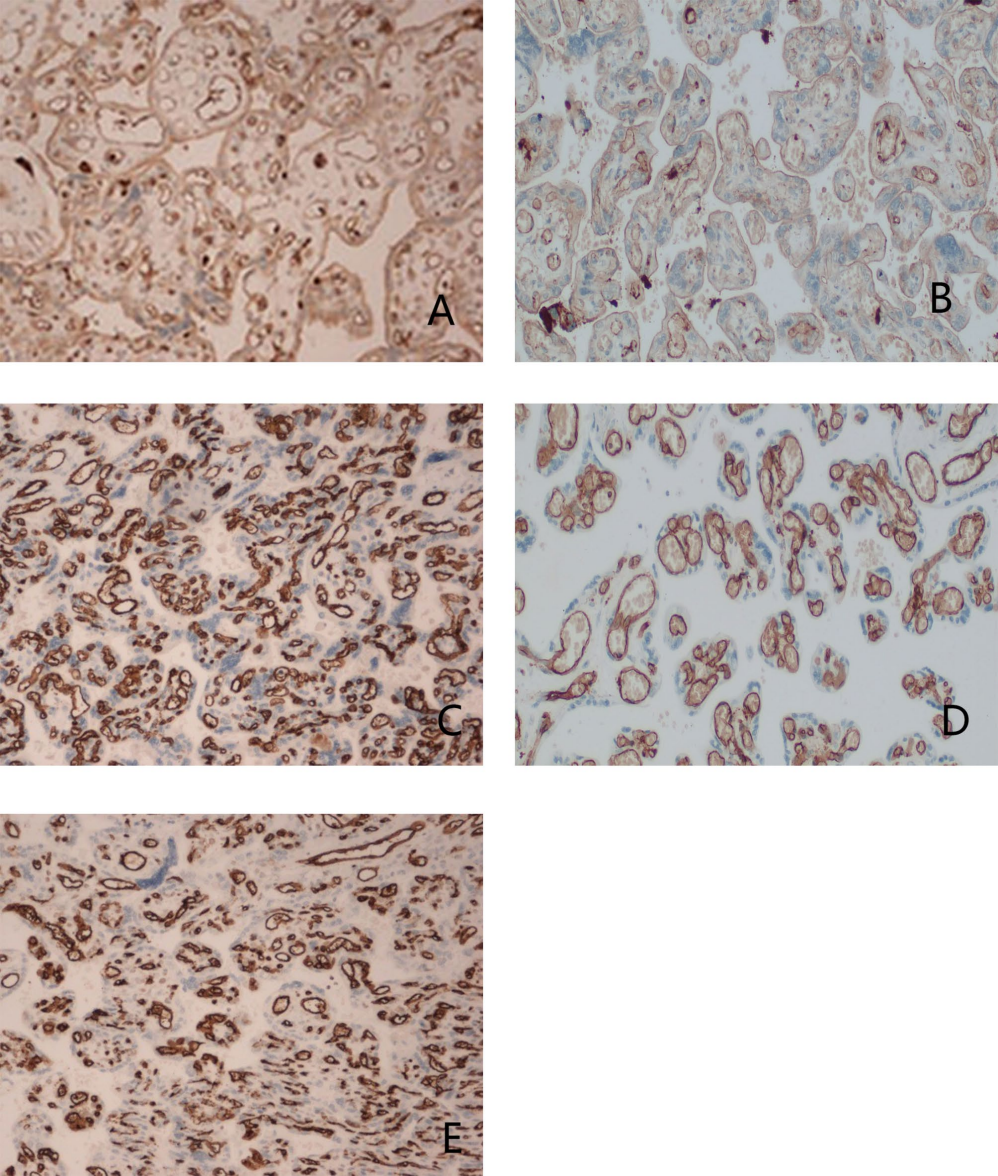
Representative immunohistochemical stains of placenta in our case. VEGF immunohistochemical staining of the massive FMH placental tissues in our cases (**A**) and of the healthy placental tissue (**B**), CD34 immunohistochemical staining of the massive FMH placental tissues in our cases (**C**) and of the healthy placental tissue (**D**), CD31 immunohistochemical staining of the massive FMH placental tissues in our cases (**E**).(200X original magnification)

Immunohistochemical staining revealed that VEGF, CD34, and CD31 expression was seen in both cases. The staining confined to the endothelial cells of the capillaries was diffusely positive, which involved approximately 50% of the placental villi volume in some areas. Representative pictures of the aforementioned findings are presented in Fig. 2a-e.

## Discussion and conclusions

Massive fetomaternal hemorrhage is a rare and serious event. Most spontaneous massive FMHs occur unexpectedly and are clinically silent. Therefore, the prenatal diagnosis of FMH is difficult, and studies have proved that the morbidity of FMH is highly underestimated because of the underutilization of confirmatory FMH test [8]. Presenting signs and symptoms of significant FMH usually include decreased or absent fetal movements, hydrops fetalis, and non-reassuring fetal tracing [9]. Reduced fetal movement may be the only complaint, but is nonspecific for FMH [10]. Studies have reported a link between intrauterine growth restriction and massive FMH as in case 2 [11]. Also, the infant’s pallor is a subtle but important sign of massive FMH [12]. Through detecting the presence of fetal blood cells and molecules derived from the fetus in maternal circulation, FMH can be confirmed and quantitatively assessed using laboratory techniques such as the Kleihauer-Betke test, flow cytometry and liquid chromatography [12]. Intrauterine blood transfusion can correct fetal anemia of prenatal diagnosed FMH but is unable to stop ongoing bleeding and may require repeat intrauterine blood transfusion [3]. The perinatal prognosis of FMH may improve via treatment with prompt delivery by caesarean section and neonatal blood transfusion [13].

Since most cases of FMH occur transplacentally, the placenta is thought to demonstrate gross and microscopic changes that are associated with FMH. The current study found that some placental pathology abnormalities such as parenchymal pallor increased nRBCs and SKs in placentas with FMH. A healthy placenta is capable of controlling dissolved nutrients and gas exchange and acts as a natural selective barrier between the maternal and fetal blood circulations. Macroscopically, pallor of the placental parenchyma tends to occur in cases of FMH in which a large volume of blood is lost. Pallor is associated with decreased perfusion of the chorionic villi followed by fetal placental blood loss or decreased blood flow. It has been reported that the placental pathologies of most massive fetomaternal hemorrhage cases were acute-onset placental lesions [14]. Acute FMH occurs when a large volume of fetal blood is suddenly transferred into the maternal circulation, which may lead to hemodynamics changes in the placenta, such as thrombosis and vasoconstriction. In contrast, chronic FMH is often less visible on placental examination, as the bleeding occurs over a longer period of time and may be more diffuse. In some cases, the placenta may become swollen and edematous, resulting in increased placental weight. The weight of the placenta was in the normal range in both cases because these were acute events and lacking chronic placental abnormalities.

According to the classification of placental lesions, placental pathology is subcategorized into vascular, inflammatory-immune, and other disorders [15]. Fetomaternal hemorrhage happens when the smaller vessels in the distal villi erupt, causing a large volume of fetal RBCs from the higher pressure fetal blood vessels to escape into the intervillous space and peripheral maternal circulation [16]. One study reported that acute FMH was associated with chorangiosis, which is characterized by vascular proliferation in the placental villi [17]. In contrast, chronic FMH was associated with villous fibrosis and decreased villous vascularity [18]. Previous study had found that the degree of villous fibrosis was significantly higher in cases of severe FMH compared to mild or moderate cases [18]. Therefore, the severity of FMH may be correlated with the extent of placental pathology. Intervillous thrombi (IVT) are considered one of the typical placental histologic findings associated with FMH [9]. These IVTs represent multilayered hemorrhages containing maternal and fetal blood. When the maternal and fetal blood types are compatible, there is a relative lack of IVT pathologic changes [19]. Therefore, it has been hypothesized that the maternally activated clotting system resulting from ABO incompatibility may be a protective factor for the extension of massive fetal hemorrhage. Increased nRBCs in the placenta have been reported to be positively associated with higher volumes of fetomaternal hemorrhage [7]. Severe and chronic blood loss may cause compensatory hemopoietic mechanisms that result in larger nucleated cells trapped in the gas-exchanging placental villi [17]. The relatively mild increased fetal nucleated red blood cells in our study may be attributed to the acute loss of fetal blood. The subsequent high output heart failure can result in edema of the placental villi accompanied by hydrops fetalis [20].

**Fig. 3:**
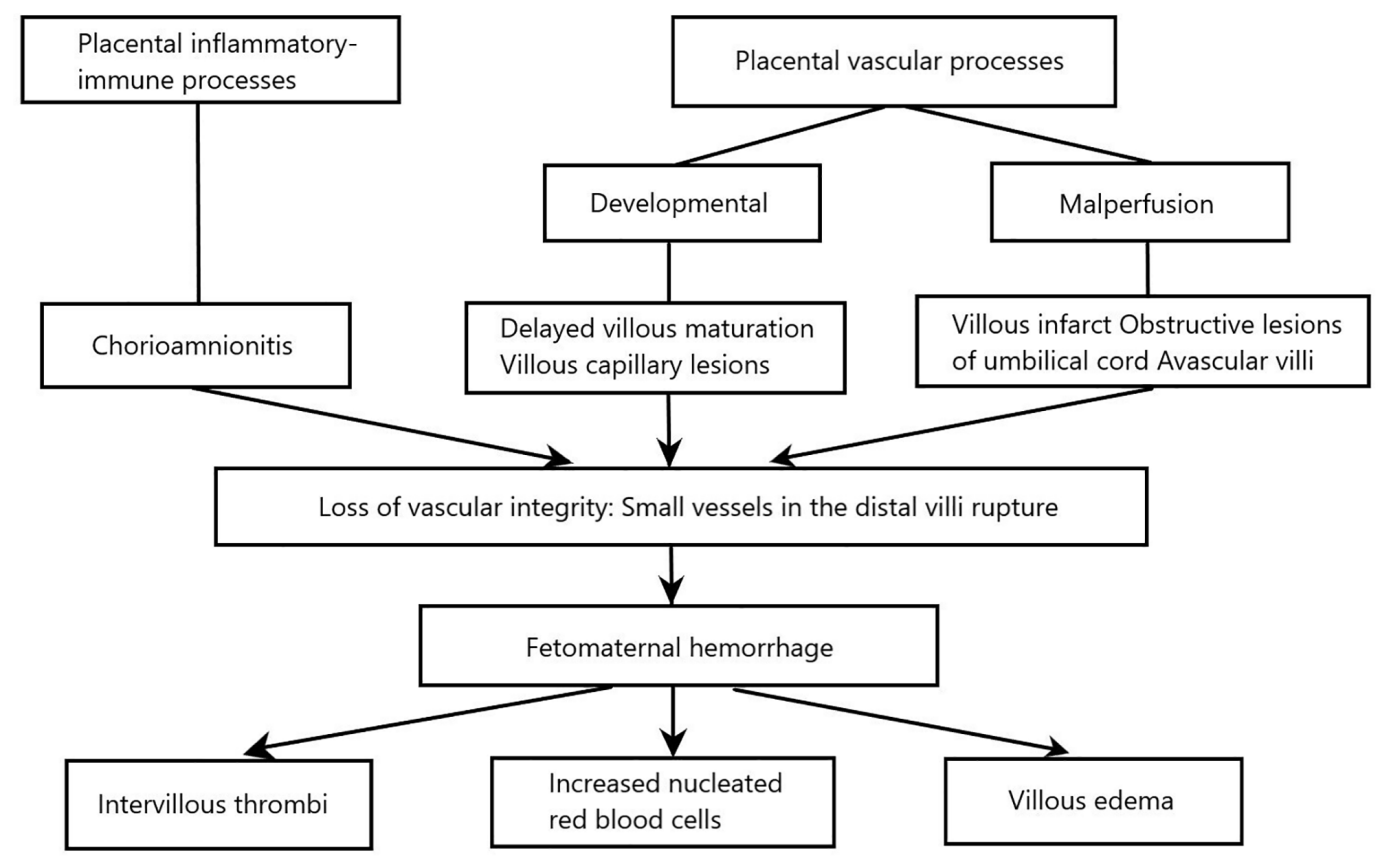
Classification of placental lesions related to massive fetomaternal hemorrhage

However, many questions remain regarding the cause of fetal vascular integrity loss lesions and the factors that influence the size or duration of hemorrhage episodes. It has been demonstrated that the risk of FMH might increase under conditions of placental stromal-vascular developmental or malperfusion lesions such as parenchymal infarcts [18], avascular villi [9], villous immaturity [12], and umbilical vein thrombosis [21]. Villous capillary lesions including chorioangiomas or choriocarcinoma have also been identified as causes of internal abruption [22]. In our cases we saw increased syncytial knot density, a factor whose pathophysiological mechanism in FMH has not yet been specifically described. The function of the SKs was considered as a response to trophoblastic ischemia that in turn causes impaired maintenance of fetomaternal exchange and contributes to intrauterine fetal distress [23]. In addition to the vascular pathologic features, uteroplacental inflammation that can damage the trophoblasts may play a role in FMH [24]. The abnormal placenta findings are summarized in Fig. 3.

The aforementioned structural changes in turn cause the functional disturbance of the placenta and may be associated with hypoxic conditions. Angiogenesis is considered a crucial process responsible for the correct function of the placenta [25]. Vascular endothelial growth factor (VEGF) is a major mediator of endothelial cell angiogenesis that can determine the level of villous vascularization and the formation of terminal villi [26]. CD34 has been widely used as a vascular endothelial progenitor [27]. CD31 is a panendothelial marker expressed in the blood and lymphatic vessels [28]. These markers play important roles in angiogenesis and have not been frequently evaluated or reported in the literature regarding FMH. The expression of VEGF, CD34, and CD31 that stained the newly formed vessels in our cases was a feature that may be induced by hypoxia. Increased endothelial VEGF can cause disruption of the placenta barrier resulting in fetoplacental vascular leaks [29]. In general, placental lesions that increase the angiogenesis of fetoplacental vessels are followed by increased risk of maternal-fetal hemorrhage because of their tendency to change the permeability of the maternal-fetal barrier.

If placental abnormalities in the structure or function act as a pivotal part of the etiology of FMH, the investigation of key placental features and their mechanisms could be a useful tool for determining the cause of FMH. However, understanding the significance of specific lesions is complicated by problems regarding the standardization in placental diagnosis, reporting practice, and clinical translation in clinical studies [30]. The precise roles of the various placental lesions in FMH remain uncertain, and most of these lesions may not necessarily be considered pathognomonic of FMH. For example, IVT has been related to fetal growth restriction and gestational diabetes [9, 31]. Other mechanisms also lead to IVT, such as trophoblastic apoptosis, clotting factors, and increased blood viscosity. Another study of FMH placenta suggested that IVT is not associated with FMH volume [19]. In addition, increased fetal nRBCs in the term placenta are associated with maternal diabetes, fetal anemia, and congenital TORCH infections [32]. Perinatal fetal RBC trafficking into the maternal circulation is likely to occur in women with complications such as pre-eclampsia and placenta previa because of placental dysregulated function in the interface between fetal and maternal circulation [33]. However, some studies have reported that most cases of massive FMH occur in the absence of any clinical risk factors [34]. The placentas of women with massive FMH show no pathologic findings on either gross or microscopic examination [35]. It is also difficult to interpret that the histopathological lesions of placenta occur prior to FMH because most placental lesions associated with fetal hypoxic conditions arise due to FMH.

In conclusion, the importance of considering massive fetomaternal hemorrhage as a potential cause of non-reassuring fetal status should not be underestimated. The findings of this study suggest that the presence of parenchymal pallor, increased nucleated red blood cells, and syncytial knots in the placenta, along with VEGF, CD34, and CD31 expression in the endothelial cells of the capillaries, are characteristic of massive FMH placenta. In future studies, basic and clinical scientists should combine their efforts to understand the specific origins of placental lesions or features that indicate the lesions are responsible for FMH. This could ensure that the implications of histopathological observations in FMH can be fully appreciated.

## Data Availability

The case reports contained clinical data from the medical records in The Second Affiliated Hospital of Wenzhou Medical University. Additional information is available from the corresponding author upon reasonable request.

## Abbreviations

FMH: fetomaternal hemorrhage
nRBCs: nucleated red blood cells
SKs: syncytial knots
H&E: Hematoxylin and eosin
VEGF: vascular endothelial growth factor
HbF: hemoglobin F
KB: Kleihauer-Betke
IVT: Intervillous thrombi
